# Successful Combined Treatment of Giant Carotid Body Tumor with Embolization Applied before Surgery

**DOI:** 10.3400/avd.cr.21-00011

**Published:** 2021-06-25

**Authors:** Hakan Usta, Izatullah Jalalzai, Ferhat Borulu, Eyupserhat Calik, Bilgehan Erkut

**Affiliations:** 1 Atatürk University, Medical Faculty, Department of Cardiovascular Surgery, Erzurum, Turkey

**Keywords:** giant carotid body tumor, combined treatment, embolization

## Abstract

Carotid body tumors are defined as unusual tumors of neuroectodermal origin that occur in the carotid bifurcation. These generally benign masses grow slowly; then, they become symptomatic with enlargement. In this study, we present a case of a 66-year-old female patient diagnosed with a carotid body tumor with a diameter of 8×9×10 cm. The patient was surgically treated 2 days after embolization due to the wideness of the mass and surgical comorbidity. Furthermore, this article puts emphasis on the importance of embolization before curative surgery in carotid body tumors with large and high blood supply.

## Introduction

Paragangliomas are described as neoplasms that originate from paraganglia cells of the autonomic nervous system. They can be found primarily in the head and neck region, along the carotid body, jugular foramen, vagus, glossopharyngeal nerves, and middle ear. Characteristically, they are often located in the carotid body at the bifurcation of the common carotid artery and can invade the carotid system branches.

Surgical resection is the standard treatment practice for carotid body tumors. However, surgical treatment often carries a risk in terms of bleeding, cranial nerve damage, and stroke in large tumors with high blood supply. In recent years, preoperative embolization has been recommended to lessen possible complications in large tumors.^1,2)^ In this study, we performed embolization to reduce blood flow to the giant carotid body tumor in the preoperative period and removed the tumor using successful surgical treatment afterward.

## Case Report

**Figure 1:**
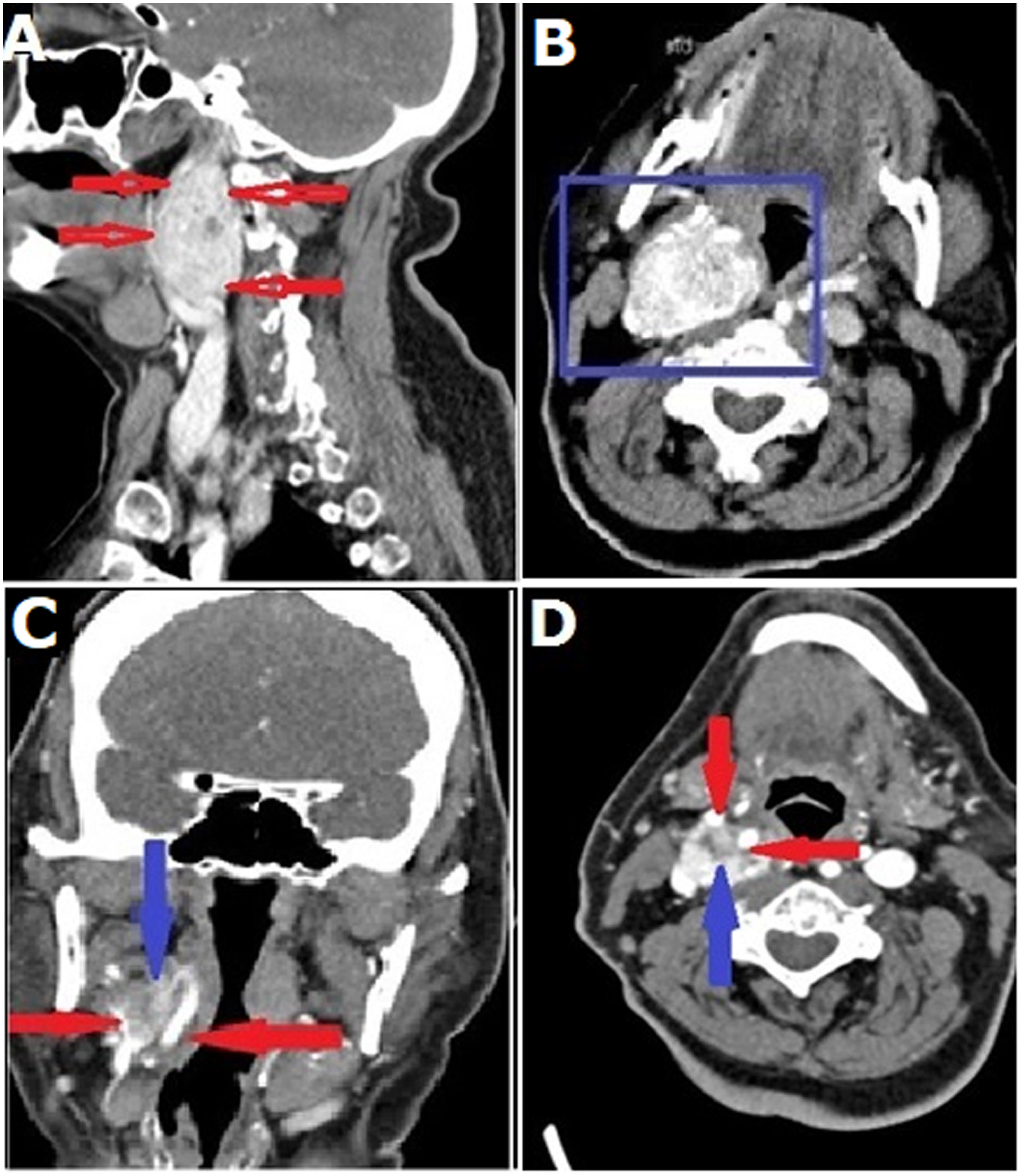
Fig. 1 Contrast-enhanced computed tomography (CT) with early arterial phase contrast shows a heterogeneously enhancing mass at the right carotid bifurcation. (**A**) The sagittal section depicts a giant carotid body tumor causing bending of the branches of the carotid artery (red arrows). (**B**) In the axial section of CT angiography, there is an image of the internal and external carotid arteries pushed due to the presence of the tumor (blue frame). (**C**) In the coronal section of the neck CT angiography taken 2 days after embolization, it can be seen that the intense contrast retention of the tumor formation decreases, the mass is reduced, and the internal-external carotid artery branches are clearly prominent (red and blue arrows). (**D**) It was found that intense contrast enhancement decreased in the tumor formation image, the mass became smaller, and the internal-external carotid artery branches were clearly seen in the axial sections (red and blue arrows).

**Figure 2:**
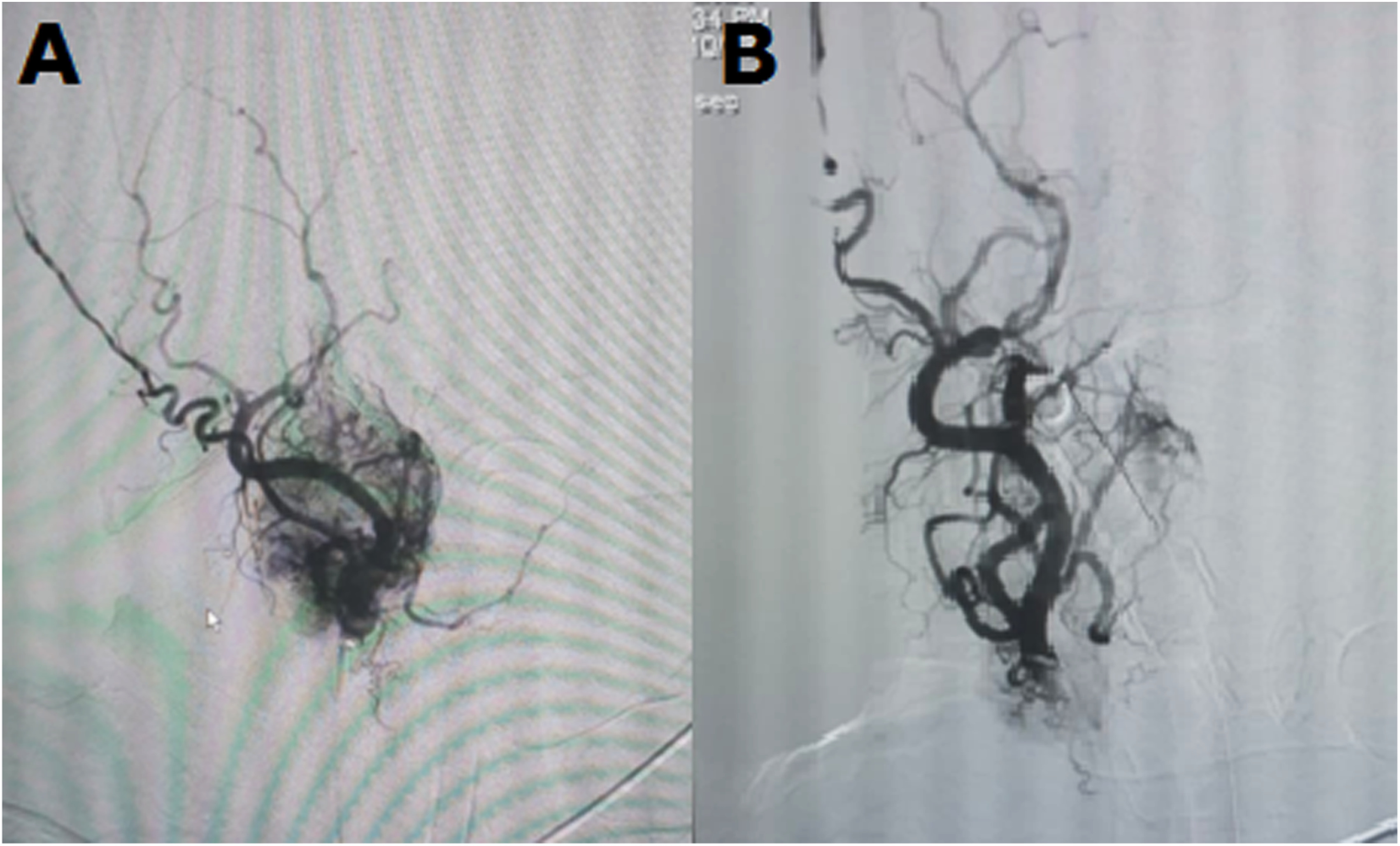
Fig. 2 Digital subtraction angiography was performed after obtaining the informed consent form. It was observed that the lesion was fed with multiple collaterals originating from the carotid artery branches, and the lesion was suitable for embolization (**A**). Following the selective catheterization of the carotid artery branches in embolization, all the feeding arteries were entered one by one, and the vessels belonging to the tumor were tried to be closed with Onyx injection. Particles were given slowly by continuous fluoroscopy to prevent the backflow of particles. The tumor was largely devascularized with embolization (**B**).

**Figure 3:**
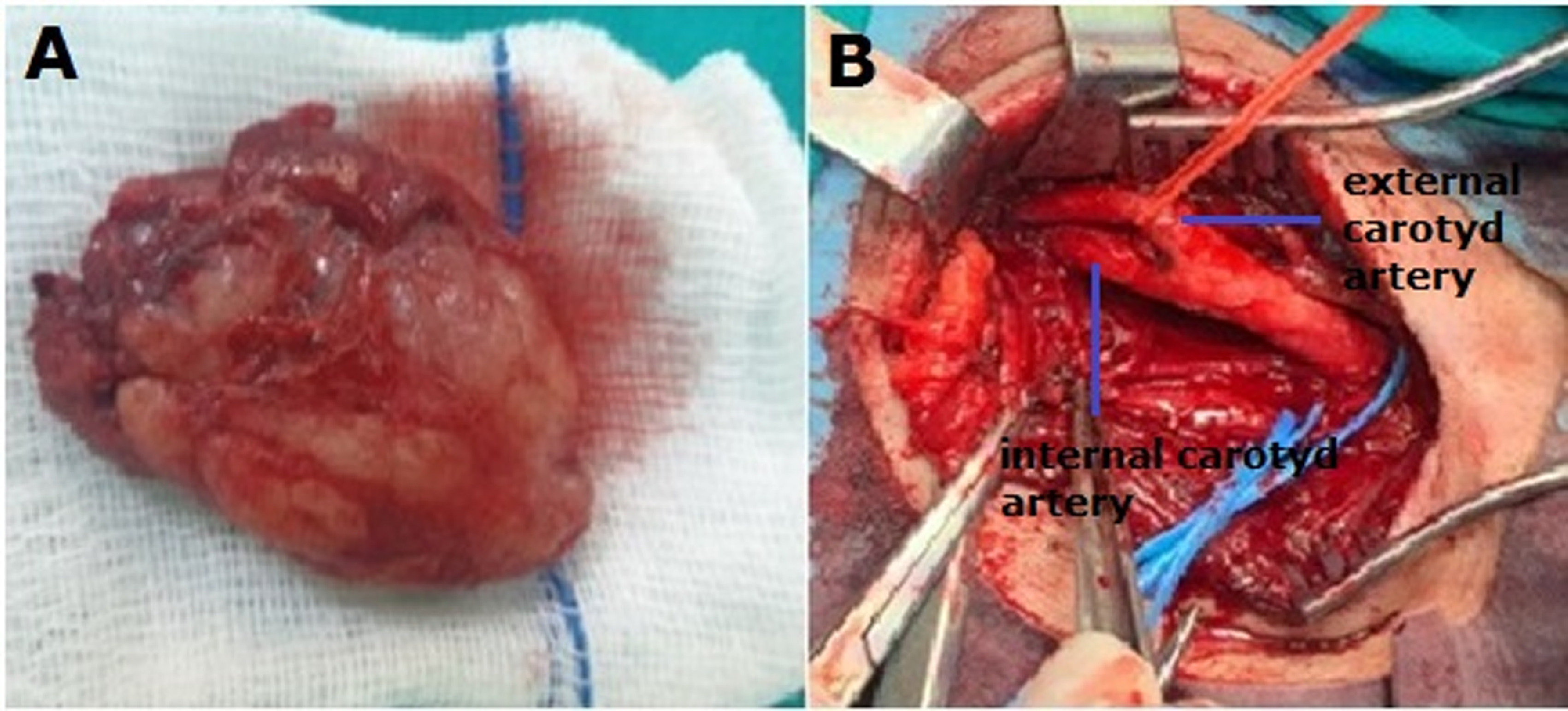
Fig. 3 The mass was reduced by embolization of the branches of the internal and external carotid artery feeding the mass (**A**). The carotid tumor was removed in a short time without excessive bleeding and damage to the nerves, and the carotid arterial system was easily exposed (**B**).

A 66-year-old female patient was admitted with the diagnosis of an approximately 10 cm painless and immobile mass between the clavicle and the chin on the right side of the neck. A vascular mass originating from the carotid body was detected in ultrasonography and a tomography was performed. In sagittal and axial image of contrast-enhanced computed tomography (CT), a solid lesion in the right carotid area was detected that pushes the internal and external carotid artery and jugular vein; it was approximately 80 mm×90 mm×100 mm in size, with smooth contours and well-circumscribed, intense contrast enhancement (**Figs. 1A** and **1B**). Embolization was decided before curative surgery as the large diameter of the mass made it difficult for dissection due to compression on surrounding nerves, and dense collaterals that could cause bleeding. Digital subtraction angiography was depicted that the lesion was fed with multiple collaterals originating from the external and internal carotid arteries and the lesion was suitable for embolization (**Fig. 2A**). In embolization, following the right external and internal carotid artery selective catheterization, all the feeding arteries emerging from these vessels were entered one by one and the vessels belonging to the tumor were tried to be closed with Onyx® glue-cast particles (an adhesive liquid embolic agent) under general anesthesia and subtracted fluoroscopic monitoring. Particles were introduced slowly by continuous fluoroscopy to prevent the backflow of particles. The procedure resulted in a significant decrease in blood flow and the tumor was observed to shrink in digital subtraction angiography (**Fig. 2B**). No complications were noted to occur during and after the procedure. Neck contrast-enhanced CT performed after embolization showed that the intense contrast enhancement decreased, the carotid artery branches became prominent and their arching decreased in coronal and axial images (**Figs. 1C** and **1D**). The carotid body tumor in our case was type II, as per the classification made by Shamblin et al.^3)^ Two days after embolization, the patient was taken to surgery for curative tumor excision. The tumor was dissected without damaging the internal carotid artery, and it was removed completely (**Fig. 3A**). After the tumor was removed, the anatomical structure of the carotid artery became fully visible (**Fig. 3B**). During the operation, residual arterial tortuous structures were seen around the left nervus vagus; all of these vascular structures were dissected while protecting the nerve. There was no nerve damage. The patient had mild swallowing difficulty in the postoperative period; however, it improved after 1 month, without recurrence.

## Discussion

Paragangliomas constitute 0.6% of head and neck tumors.^4)^ Approximately, 80% of all paragangliomas are either carotid body tumor or glomus jugulare tumor. Carotid body tumors are usually asymptomatic and grow slowly. As it grows, it can involve one or more of the carotid artery and its branches without narrowing it. As the tumor progresses, there may be involvement in the lower cranial nerves and adjacent pharynx.^5)^

Symptoms that can be observed such as dysphagia, voice changes, cough, tinnitus, hearing loss, headache, dizziness, bradycardia, syncope, vocal cord paralysis, sympathetic nerve involvement, Horner syndrome, and compression of the hypoglossal, glossopharyngeal, vagus nerves and sympathetic chain; these can all be related to the invasion.^6)^

The location of the tumor and its vascular origin can be demonstrated with color Doppler ultrasonography. Treatment planning is made by determining the exact localization and extension of the lesion via contrast-enhanced CT or magnetic resonance imaging. Paragangliomas are stained homogeneously and intensely with contrast material on CT. Digital subtraction angiography is important to view the nourishing arterial anatomy and collaterals and to examine the internal carotid artery invasion, which occurs with constriction and irregularity.

Based on Shamblin’s classification,^3)^ carotid body tumors are classified into three types. Carotid body tumors with higher Shamblin grade tends to have more severe neurological complications upon surgery,^7)^ mostly cranial nerve injuries.^7,8)^ Preoperative embolization can be performed to reduce intraoperative bleeding by reducing the size of the tumor with vascularization, shorten the duration of the surgical procedure, decrease the rate of cranial nerve injury, and increase the possibility of complete removal of the lesion.^9,10)^ One recent report compared Shamblin class II and III carotid body tumors treated with preoperative embolization. A trend was shown in the embolization group for fewer transient ischemic attacks and cranial nerve injuries.^5)^ In a study in which 29 patients were included, preoperative embolization was performed in 17 of the 36 paragangliomas, and it was reported that embolization reduced blood loss and facilitated tumor removal.^1)^ In order for the embolization to be fully successful, all arteries feeding the tumor must be covered. For this purpose, polyvinyl alcohol, Onyx gel, liquid embolic material, isobutyl-2-cyanoacrylate and lipiodol mixture, and absorbable embolic materials can be used. Tumor size can be reduced by 25%–50% via embolization. During and after the procedure, an approximately 70% reduction in tumor blood supply is achieved by embolization without any complications.^7–9)^ Less manipulation is required in the operation and theoretically, blood loss is reduced. In our case, since the blood supply in the tumor was reduced by embolization, bleeding was easily controlled during operation, and less vessel dissection and cauterization were required. The time between embolization and surgery should be 1 to 2 days to allow the edema that has developed due to embolization to resolve; however, it should not exceed 2 weeks to prevent recanalization of the vessels.^10)^

## Conclusion

As a result, preoperative embolization in large carotid body tumors causes the tumor mass to shrink significantly, and the carotid artery branches and peripheral nerves become more prominent, resulting in reduction of complications with a comfortable surgery in a shorter time. Surgical resection is usually the definitive treatment for carotid body tumors. However, microscopic residue may remain and recurrence may occur. Therefore, patients should be followed up for a long time.
